# Dr. Paris on Poisons. [Medical Jurisprudence]

**Published:** 1824-09-01

**Authors:** 


*Medical Jurisprudence.* By Dr. PARIS and Mr. FONBLANQUE.

### TOXICOLOGY.

IN our 15th Number we concluded our second analytical article, with some observations on medical evidence, in cases of poisoning, reserving for the present paper a more particular detail of the classification, and of the history of individual poisons. Toxicology, we conceive, is one of the most important and difficult subjects of medical jurisprudence, and we acknowledge, with pleasure, our conviction that there are few people, in this country, more capable of doing justice to the difficult task than Dr. Paris. His tried abilities as a chymist and pharmacologist, eminently qualify him for this undertaking, and we cheerfully bear testimony to the able manner in which he has dicharged his duty, considering the narrow limits within which he was confined.

If we define poisons to be certain substances which, when taken in a certain quantity, destroy life, it will be very difficult to say where poisons end, and food or medicine begins. That the scale of poison runs pretty low, may be inferred from a paper in the Associated Apothecaries' Transactions, where we have instances of poisoning by an orange, by pickled pork, and by a mutton chop. We think, however, that this is carrying the thing too far—and that the term poison should be limited to those active and deleterious substances which, *in small quantities*, destroy life, whether taken by mistake or administered by design. We know indeed that it would be difficult to make this definition apply in all cases—but it is better to have some definition to restrain us than the wild and unbridled latitude assumed in the paper above alluded to.

The classification of poisonous substances has been a matter of much difficulty and speculation, according to the botanical, chemical, or pathological views of authors. It is perhaps impossible to meet or combine all these views in any one system of arrangement, but Dr. Paris (for we need hardly include Mr. Fonblanque's name in this department) has endeavoured to select one which may approach the nearest to the imaginary fabric of perfection, viz. that proposed first by Foderée and adopted by Orfila, having pathology for its basis, but yet distributing the different poisons (with some few exceptions) in an order corresponding with that of their natural history. Dr. Paris, therefore, gives the following sketch of an arrangement originally proposed in the 5th edition of his Pharmacologia.

"A CLASSIFICATION OF THE DIFFERENT MODES BY WHICH POISONS PRODUCE THEIR EFFECTS.

1. BY ACTING THROUGH THE MEDIUM OF THE NERVES WITHOUT BEING ABSORBED, AND WITHOUT EXCITING ANY LOCAL INFLAMMATION.

a. *By which the functions of the nervous system are destroyed.*

| | | | |
|---|---|---|---|
| *Acrid.* | Aconite. Jatropa Curcas | *Narcotic.* | Essential Oil of Almonds.† Camphor.† Opium.† ? |
| *Narcotico-Acrid.* | Alcohol. Oil of Tobacco. | | |

b. *By rendering the heart insensible to the stimulus of the blood.*

Infusion of Tobacco.
*Upas Antiar.*

---

"† This mark denotes that the substance, against which it is placed, may also act by being absorbed."

II. BY ENTERING THE CIRCULATION, AND ACTING THROUGH THAT MEDIUM WITH DIFFERENT DEGREES OF FORCE, ON THE HEART, BRAIN, AND ALIMENTARY CANAL.

| | | | |
|---|---|---|---|
| Corrosive. | Arsenic.<br>Emetic Tartar.<br>Muriate of Baryta. | Narcotic. | Opium.‡<br>Lettuce.<br>Henbane.<br>Prussic acid. |
| Acrid. | Hellebore.<br>Savine.<br>Meadow Saffron.<br>Squill. | Narcotico Acrid. | Deadly Nightshade.‡<br>Hemlock.<br>Camphor.‡<br>Cocculus Indicus. |

III. BY A LOCAL ACTION ON THE MUCOUS MEMBRANE OF THE STOMACH, EXCITING A HIGH DEGREE OF INFLAMMATION.

| | | |
|---|---|---|
| Corrosive Sublimate.†<br>Verdegris.<br>Muriate and<br>Oxide of Tin.<br>Sulphate of Zinc.<br>Nitrate of Silver.<br>Acids.<br>Alkalies.<br>Cantharides.† | Acrid. | Bryony.<br>Elaterium.†<br>Colocynth.†<br>Camboge.<br>Euphorbium.<br>Hedge Hyssop.<br>Croton Tiglium.<br>Ranunculi." 207. |

The first class comprehends such poisons as operate, through the medium of the nerves, upon the organs directly subservient to life—and therefore it is not absolutely necessary that they should be introduced into the stomach, being capable of exerting their deleterious influence by application to any part duly supplied with nerves. The second class acts, or is supposed to act, only through the medium of the circulation. We can scarcely think that the action of prussic acid and alcohol, or essential oil of almonds, is so different as to entitle them to different classes. Surely a dose of prussic acid that destroys life in a few seconds must act more through the medium of the nerves than the blood-vessels; and yet it is placed among the substances whose action is through the latter channel. Again, as arsenic, emetic tartar, hellebore, squill, &c. must have a powerful local action on the delicate structures of the stomach, it seems unnatural to separate them from mercurials, cantharides, &c. Hippolitus Cloquet, a recent French writer on toxicology, (43d vol. Dict. des Sciences Med.) places both the second and third classes of Dr. Paris, under the head of "irritant, corrosive, or escharotic poisons."

The mineral poisons, being the substances generally employed

---

" ‡ Signifies that the article has also a local action."

as the instruments of crime, are first treated of by Dr. Paris, by examining their external characters; 2d, their chemical composition; 3d, their tests; 4th, the symptoms; 5th, physiological action; 6th, forms of application; 7th, lesions of structure produced; 8th, phenomena post mortem.

1. *Arsenic.* This substance combines with two proportions of oxygen, forming arsenious and arsenic acids—the former, or white oxide, as it is called, is the most fatal of all the mineral poisons, and the most frequently employed by the assassin and suicide. Dr. Paris denies the alliaceous or garlic smell commonly attributed to the vapour of arsenic, affirming that when such smell is emitted the oxide is reduced to its *metallic* state. Dr. Paris does not follow his own plan in the consideration of arsenic. The *tests*, instead of coming in under the 3d head, are not treated of till the last. This, however, is of very little consequence.

*Symptomatology.* Dr. Paris divides the symptoms of poisoning by arsenic into three classes, or degrees of intensity —1st, where the lesion, though dangerous, is not fatal—2d, where death does not take place till after 24 hours—3d, where the poison proves fatal within the above space of time. The symptoms indicative of the *first* degree of intensity are, uneasiness of the præcordia, cholic, soreness of the gums, itching of the surface, head-ach, strangury, &c. Occasionally vomiting is produced, with dryness and heat of the fauces. Though life be spared under such circumstances, a train of consecutive symptoms may harrass the patient for a long period. In the *second* degree of intensity, where the patient lives two, three, or more days, the earliest symptoms are heat, thirst, vomiting, inexpressible anxiety, purging or tenesmus, wandering pains, quick feeble pulse, head-ache, abdominal distention, and, towards the close of the scene, sleeplessness or convulsions, and sudden death at last. In the *third* degree, we have austere taste, heat and constriction of the pharynx and œsophagus, excruciating pains in the stomach and bowels, with vomiting of brownish or other discoloured matters, not unfrequently mixed with blood, inexpressible anxiety about the præcordia, faintings, dark and fetid evacuations from the bowels, unquenchable thirst, palpitations, cramps, strangury, laborious respiration, cold sweats, hiccup, occasionally convulsions, but rarely delirium, death. These symptoms will not all be found or rarely in the same person, and therefore the absence of several of them is no proof that arsenic has not been administered.

*Modes of Poisoning.* Life may be destroyed by the internal administration of arsenious acid, or by its external application to abraded surfaces, the symptoms being analogous in both cases—but often more rapid in the latter than in the former mode. Mr. Brodie tried many experients on animals, taking care that they should not be able to lick the wounds where the arsenic was applied, and the results were uniform. The stomach was, in every case, not only more violently inflamed than when the poison had been internally administered, but this inflammation preceded any inflammatory appearance in the wound.

*Physiological Action.* In the Philosophical Transactions for 1811, Mr. Brodie has shown, by experiments, that the deleterious influence of arsenious acid arises from its being absorbed —and consequently that it must be regarded as a vital rather than a chemical agent. In most of these experiments death took place too soon to be the consequence of local inflammation of the stomach—where indeed the marks of phlogosis were sometimes scarcely visible—excepting as the effects of the poison in the general circulation. It sometimes happens, however, that death will ensue from the local action of arsenic in the stomach, the animal having survived the effects produced on the organs more immediately subservient to life, as the brain and heart. This may be termed "*consecutive poisoning.*"

*Organic Lesions, post mortem.* These are not uniform. The stomach will generally be found more or less inflamed, with patches and streaks of different colours—the villous coat softened—ulceration only in protracted cases—the appearance of sloughs fallacious, as they are usually only layers of effused blood—thickenings of the coats in some instances. The duodenum generally exhibits analogous lesions, and the whole line of the alimentary canal will be found more or less affected, according to the quantity of arsenic that has been administered and other circumstances. It is curious that the rectum appears sometimes to suffer more than any higher tract of intestine. Mr. Brodie never found the pharynx or œsophagus inflamed, but many others have given different testimony. The parts of generation, in both sexes, have not very unfrequently been found gangrened or swelled. Brodie, Ruysch, Jaegar, agree that the blood is not coagulated in the vessels, where death has been produced by arsenic. Our author properly cautions the anatomist not to confound the red or violet colour of inflammation with that which is occasionally found to arise from the ingestion of certain coloured drinks. Infusion of the red poppy has done this—tincture of cardamoms will also produce the same effect.

5. *Detection of Arsenious Acid.* This section being merely an amplification of what Dr. Paris has published in his Pharmacologia, we need not—indeed we could not give a satisfactory analysis of it here. The work alluded to is now so universally diffused, that his sentiments are well known. Dr. Paris is of opinion that the reproduction of the metal is not necessary as an unequivocal proof of the presence of arsenious acid. He thinks the silver and copper tests, described in both his works, are capable, under proper management and precaution, of furnishing striking and infallible indications—even more satisfactory in their results than the metallic reproduction. In order to preserve some continuity in the chain of our analysis, we shall give the two tests abovementioned, as they occupy but a very small space.

" *Fused Nitrate of Silver, or Lunar Caustic.* For this test we are indebted to *Mr. Hume*, who first suggested its application in the Philosophical Magazine for May 1809, (vol. xxxiii.) His method of using it is as follows: into a clean Florence flask introduce two or three grains of the suspected substance, in the state of powder, to which add about eight ounces of rain or distilled water, and heat the solution until it begins to boil; then while it boils frequently shake the flask, and add to the hot solution a grain or two of sub-carbonate of potass, agitating the whole to make the mixture uniform. Pour into a wine glass about two table spoonsful of the solution, and touch the surface of the fluid with a stick of lunar caustic. If arsenic be present, a beautiful yellow precipitate will instantly proceed from the point of contact, and settle towards the bottom of the glass as a flocculent and copious precipitate. By this test the 60th part of a grain may be satisfactorily recognized in two ounces of water. The presence of some alkali is essential to the success of the experiment, since arsenious acid is incapable, by the operation of simple affinity, to decompose the *nitrate of silver*.*" 240.

Objections to the above have been made, and they have been

"* If any trifling opacity occur in a simple solution of arsenic, when assayed by the nitrate of silver, it may be considered as the effects of some casual impurity; this may be farther demonstrated by bringing over the surface of the arsenical liquid, a piece of blotting paper, or a stopper moistened with a solution of ammonia, when there will instantly form a copious yellow precipitate of arsenite of silver. If this experiment be performed by spreading the mixed solutions of arsenious acid and nitrate of silver over a surface of glass, laid upon white paper, the result will be most striking and beautiful, for, on slowly bringing the ammoniacal test over it, the yellow cloud will gradually diffuse itself over the surface."

answered by Dr. Paris. The modification of the experiment which he recommends is, to drop the suspected liquor on a piece of white paper, making with it a broad line—along which line a stick of lunar caustic is to be slowly drawn several times successively, when a streak is produced, of a colour resembling that of Indian yellow. This is equally produced by the presence of arsenic and that of an alkaline phosphate—but the one from the *former* is rough, curdy, and flocculent, as if effected by a crayon—that from the *latter*, is homogeneous and uniform, resembling a water-colour laid smoothly on with a brush. A still more important distinction soon succeeds. In less than two minutes, the phosphoric yellow fades into a *sad green*, and becomes gradually darker—ultimately, quite black; while the arsenical yellow, on the contrary, remains permanent, or nearly so, ultimately turning brown. The sunshine must be avoided in this experiment.

" *Sulphate of Copper*. This test of arsenic is the one discovered by *Scheele;* when added to the *arsenite of potass* a beautiful green precipitate (constituting a pigment known by the name of *Scheele's green)* is produced; 'so decidedly,' says *Dr. Bostock*, 'does this phenomenon indicate the presence of arsenic, that I thought it desirable to ascertain, as exactly as possible, what were the best proportions in which the ingredients should be employed, and in what way they should be mixed, so as to exhibit the effect in the most obvious manner. After a number of trials, in which the substances were employed in various quantities, and under different circumstances, I am disposed to recommend that the proportions of the *arsenic*, the *potass*, and the *sulphate of copper*, should be to each other as the numbers *one*, *three*, and *five*, respectively; for instance, if one grain of arsenic and three grains of potass, be dissolved in two drachms of water; and, in another equal quantity of water, five grains of sulphate be dissolved, we have two solutions, which are transparent, and nearly colourless; but upon mixing them together, the whole is converted into the most beautiful grass-green, from which a copious precipitate of the same hue slowly subsides, leaving the supernatant fluid nearly without colour. If the same materials are employed in the same manner, but without the arsenic, a delicate *sky-blue* is formed, which is so decidedly different from the former colour as not to admit of the possibility of error.' In this experiment then, as well as in that with the nitrate of silver, it is necessary that the arsenious acid should be combined with an alkaline base; and for the same reason, in order to bring the double elective attractions into play; *Mr. Hume* has accordingly availed himself of the property of ammonia, to form an *ammoniuret of copper*, which is to be made according to the formula already given for the preparation of the silver test." 246.

For answers to the objections that have been made to this test we must refer the reader to Dr. Paris's works.

The detection of arsenic amidst a complicated mass of alimentary matter has long been considered a process of great trouble; and various proposals have been made to obviate the difficulties. Dr. Paris recommends the juridical chemist, who suspects arsenic in broth, coffee, or any coloured liquid, to add a solution of *ammoniuret of silver*, and, thus, to precipitate indiscriminately all the bodies which it may be capable of so affecting. The precipitate may then be collected, and submitted to heat in a glass tube, as before directed.

Considering the difficulties and fallacies in the way of detecting minute portions of arsenic in the human body, it evidently requires an experienced and practical chemist for conducting the process. In the hands of such a person, we should suppose the foregoing tests would be sufficient—for we imagine, that a jury of this country will very rarely place the die of a man's fate on the result *alone* of a complicated chemical process for testing the presence of arsenic. There must be very strong evidence of a moral nature to aid such proofs of criminality before a jury would pass the verdict of guilty.

The means of obviating the poison of arsenic does not properly, we suppose, fall within the range of forensic medicine, as Dr. Paris does not allude to them, though he gives the antidotes, or modes of treatment, in most of the other poisons. In his Pharmacologia, however, Dr. Paris has given sensible and interesting instructions on this point. The ejection from the stomach of the arsenical poison is the *sine quâ non*, since we know of no substitute capable of decomposing or rendering inert this deleterious agent. Lime, or its carbonate, indeed, renders arsenic less soluble; but this is a thing which no man would trust to. Hitherto emetics have been the general resource, especially the sulphates of zinc and copper; but the mechanical evacuation of the stomach by means of an elastic tube, must, we think, supersede all other means in a very short time. We conceive that it is a culpable neglect for any practitioner to be without the proper apparatus for this purpose. Lime-water, if at hand, might be injected into the stomach, as the menstruum for washing out the poison of arsenic; but this being out of the way, water alone should be immediately had recourse to for the said operation. This instrumental antidote is the only safe one—

———— quo non præsentius ullum
Auxilium venit, ac membris agit atra venena.—

When the poison has been mechanically ejected, we may then administer such remedies as promise to counteract the effects on the constitution produced by the stay of the poison in contact with the stomach.

2. *Oxymurias Hydrargyri.*—The effects of this poison will vary according as it is taken in large quantities with the view of destroying life at once, or in repeated doses so as to act as an "accumulative poison." In the former case, we have the general symptoms produced by arsenic. In the latter, salivation and its various effects. It is asserted, however, and by Dr. Paris's personal experience confirmed, that when sublimate is taken for a long time in small doses, it will induce a train of formidable symptoms, together with hectic fever—without salivation. In respect to the wonderful stories which have been related of salivation resuscitated after it had, for many months or years, ceased, and in that state, continued for years, we give no credence to them. Neither the authors that relate, nor those who quote them, take any notice of salivation without mercury, though Cullen and other nosologists, have enumerated nearly thirty species of idiopathic or symptomatic ptyalism independently of mercury. We are really astonished, that Dr. Smith and Dr. Paris should have quoted the case of Dr. Hamilton (see vol. 1 of Analytical Series, page 45) where, after a few doses of blue pill, taken *four years* previously, a salivation (mercurial) came on and lasted more than a year! Several medical Gentlemen of this metropolis have seen a lady (Mrs. Price, the relict of a surgeon in the city, and who now resides at Brighton) who for years had a copious non-mercurial ptyalism of a most distressing nature. We believe she still has it; but at all events, Dr. Armstrong and others saw her as well as ourselves, under the care of a respectable and able surgeon at Hackney—Mr. Illingworth. We lately saw another remarkable instance of this spontaneous ptyalism in a lady, attended with inordinate secretion from the whole mucous membrane of the bowels, which lasted about six years and ultimately wore her out.* If mercury committed such vagaries as these, we wonder Mr. Abernethy and others have not published some cases of the kind.

*Physiological Action.* It acts corrosively on the stomach, and sympathetically through that organ on the heart and brain, suspending their functions and producing death. When slowly administered, the case, of course, is otherwise; as it is then

* See also a case of spontaneous ptyalism related by Dr Carter in the December Number of the London Medical Repository.

absorbed into the system, and acts gradually on various structures and functions of the body.

*Antidotes and Treatment.* Plentiful dilution is the first object, and evacuation the next. This, as in the case of arsenic, should be done mechanically, as far more certain than by emetics. Whites of eggs or the gluten of flower decompose sublimate, if they are taken in large quantities—but we would infinitely prefer the mechanical evacuation.

The organic lesions discoverable afer death cannot be discriminated from those produced by other corrosive poisons.

*Detection Chemically.* Dr. Paris has entered rather more fully into the testing of sublimate in this work than in his Pharmacologia, vol II. page 241. After giving the tests from the last mentioned work, by metallization through the agency of galvanism, as recommended by Mr. Sylvester, our author goes on to state the other tests by carbonate of potash, ammonia, and lime water, succinctly given in the Pharmacologia, but here amplified, without much additional information. In forensic cases we generally have the sublimate dissolved in various coloured liquids which are liable to obscure the characteristic indications which the several reagents would otherwise occasion. In these instances Orfila recommends the previous addition of chlorine in order to discharge the colour; but many and weighty objections are made by Dr. Paris.

"It will be preferable on these occasions to precipitate the salt by an appropriate reagent, and then to assay the precipitate for metallic mercury; or to evaporate the solution, and to submit the matter so obtained to the process of sublimation, when the sublimate may be dissolved in distilled water, and examined by the tests above described. This circuitous process may, however, in many cases be rendered unnecessary, by dropping the solution on the surface of white paper, and in such a situation proceeding to its examination by tests; when the colour of the precipitate will rarely be exposed to any optical fallacy. The Galvanic process of metallic reduction will also furnish a satisfactory solution of the problem." 273.

Sublimate is very easily decomposed in the stomach by various alimentary matters, and there converted into calomel. In these cases we must seek to establish the fact, of poisoning through the detection of metallic mercury by the process of calcination and sublimation.

3. *Antimony—Tartarized.* Speaking generally, emetic substances dislodge themselves, and thus prevent fatal effects.

But this is not always the case. There are states of the system where the sensibility of the stomach (as in apoplexy, coma, &c.) is so much diminished that emetic tartar may be given in large quantities without vomiting—and here we may apprehend bad effects in the stomach, a case of which was communicated by M. Cloquet to Orfila. The symptoms produced by this salt resemble those of most other corrosive poisons, and have been described by various writers—the principal are nausea—copious vomiting—hiccup—cardialgia—pains in the stomach—colic—copious stools—syncope—convulsion, &c. death.

*Antidotes*. The great object is the discharge of the poison by vomiting, or rather by mechanical means. Strong tea, as being the nearest at hand, may be employed as the menstruum for emesis or for washing out the stomach; but no dependence should be placed on the decomposing powers of this or any other vegetable substance. Evacuation is the only sure process.

The other mineral poisons are discussed with minuteness and ability in the work before us; but we cannot follow the author through the details. We shall take some notice of another class in Dr. Paris's arrangement.

IV. *Vegetable Poisons*. These are not so important in a forensic as in a medical point of view. With the exception of opium and a few others, they must be considered as objects of accidental rather than of criminal poisoning. Even opium is far more frequently employed by the suicide than the assassin.

1. *Opium*. The primary action of this celebrated drug, when taken in considerable doses, is considered by Dr. Paris as powerfully and diffusibly stimulant; but this excitement is so very transitory as not to be apparent—the powers of life being immediately depressed, and succeeded by stupor, delirium, stertor, cold sweats, convulsions and apoplectic death.—The quantity necessary to produce these effects is purely relative—in one person it may destroy life in the quantity of a few grains—while the Turk, and opium-eater in general may take as many drachms, without inconvenience. This temporary immunity is, as may naturally be supposed, followed by proportionate suffering. Nature will not be cozened by the greatest ingenuity of man. We may borrow from her, but like a usurer she sooner or later extorts a fearful interest, and with inexorable punctuality.

The symptoms of a poisonous dose of opium are well known, and can hardly be mistaken by the youngest tyro. Insensibility, slow or stertorous breathing, livid or cadaverous countenance, cold skin, insensibility of pupils, almost imperceptible pulse—death, in from six to twenty-four hours.

*Physiological Action.* We agree with our author that opium produces death by destroying the functions of the brain, and through that the power of respiration, in consequence of which suffocation ensues. But the question is still undecided, how this destruction of the sensorial energies is affected? Dr. Paris inclines to the opinion that it is by absorption, though he does not doubt that opium may occasionally produce an effect upon the sentient extremities of the nerves of the stomach, and thus affect the brain sympathetically.

*Treatment.* The first object is the evacuation of the stomach. Here, of course, the mechanical evacuation will be more certain and expeditious than all others. If this be declined, then 15 or 20 grains of sulphate of zinc should be given; or from 5 to 10 grains of sulphate of copper dissolved in water, the vomiting to be kept up by irritation of the fauces. The affusion of cold water, by awakening the sensibility of the brain and nervous system is said to act in promoting the power of emetics. It is not, of course, till after the evacuation of opium that acids are to be given. Blood-letting, to empty the vessels of the brain, is recommended on the authority of Orfila; but as Dr. Paris ranks opium among the poisons that are absorbed from the stomach, venesection, as a powerful promoter of absorption, cannot consistently with that theory be prescribed.

*Organic Lesions Post Mortem.* Orfila asserts that no alteration can be discovered in the alimentary canal of those who have taken opium or any narcotic poison. Others, however, have observed congestions of blood in the internal organs.

*Detection.* The odour will discover the nature of the poison. The chemist may also proceed to obtain morphia from the solution.

2. *Hydrocyanic Acid.* The history and qualities of this powerful substance are sufficiently known to the public. As for antidotes there are none. The effects of the acid on the vital powers may, in some degree, be counteracted by diffusible stimuli, as brandy, ammonia, æther, camphor, &c.

3. *Belladonna.* The beautiful and tempting berries of this plant have, in numerous instances, proved fatal to children and ignorant persons in this country.

The symptoms are, extreme dryness of the mouth, fauces, and throat—difficult deglutition—dilatation of the pupil—nausea—symptoms of intoxication, with fits of laughter, ravings, violent gestures, continued motions of the hands and fingers. To these succeed redness and tumefaction of the face, feeble pulse, paralysis of the intestines, livid spots on the surface, cold sweats, convulsions—death.

*Physiological Action.* According to Orfila, this poison is absorbed into the system, and acts on the brain and nerves. At the same time it exerts a local action on the stomach—though less violent than that occasioned by the acrid poisons. That it acts sometimes purely through the medium of the nerves may be inferred from the dilatation of the pupil, by the mere application of belladonna to the eyebrow.

We must pass very rapidly over the remaining articles of the vegetable poisons, as we find but little that can lay claim to novelty.

4. *Tobacco.* Orfila supposes that the active parts of this celebrated plant are absorbed into the circulation, while the experiments of Mr. Brodie tend to prove that they operate through the medium of the nerves. It is very singular that, in Mr. B's experiments, the essential oil of tobacco was found to act very differently from the infusion—for while the *former* appeared to act exclusively on the brain, leaving the powers of the circulation unimpaired, the *latter* acted on the heart at once, suspending its action even before the animal ceased to respire, thus destroying life by syncope.

5. *Conium.* This appears to act by absorption; at the same time it exerts a local irritation, with inflammation more or less violent. After evacuation, the cerebral excitement is reduced by bleeding. The best antidote is vinegar.

6. *Nux Vomica.* The peculiar proximate principle of this substance, known by the name of *strychnia* or strychnine, is a highly alkaline salt, insupportably bitter, and, next to prussic acid, the most virulent poison known. It is supposed to exert a specific action on the spinal marrow, producing tetanus, immobility of the thorax, &c. Hence its application to paralysis, which may be considered as the reverse state of tetanus.

7. *Cocculus Indicus.* Dr. Goupil has communicated certain curious facts relating to this substance, which tend to throw some light on the poison of fishes. This substance is not only destructive to fishes, but to different carnivorous animals, and probably to man. The poisonous principle passes apparently unchanged through the organs of digestion into the absorbent system of the fishes, the flesh of the latter becoming poison to other animals.

8. *Alcohol.* This is a slow or a sudden poison. It is to its effects in the former way, that the physician must look with most anxiety. The effects of alcohol vary much according to the state of combination in which it is taken. Thus, Mr. Brande has shewn that port, madeira, and sherry, contain from one fourth to one fifth their bulk of alcohol. A man, therefore, who drinks a bottle of port daily, will take nearly half a pint of pure alcohol, which is equivalent to a pint of brandy! Were he to take the latter alone or mixed with water, the effects would be very different.

"The remote consequences too of alcohol in these different states, are as striking and distinct as their immediate effects. It is well known that diseases of the liver are most common, and the most formidable of those produced by the use of *ardent* spirits; it is equally certain that no such disorders follow the intemperate use of wine that is perfectly *pure;* let it be remembered that the greater proportion of that which is drunk in this country contains uncombined brandy, purposely added to meet the demand of the British market; and *Dr. Mac Culloch* thinks that it is to the unwitting and concealed consumption of this uncombined spirit, that we ought to attribute the prevalence of those hepatic affections which are comparatively little known to our continental neighbours. But although wine, in a state of purity, may be thus fairly excluded from the general obloquy which attaches to spirituous potations, it must not be regarded as entirely free from imputation. 'The effects of wine,' says *Rush* 'like those of tyranny in a well formed government, are first felt in the extremities; while spirits, like a bold invader, seize at once upon the vitals of the constitution.' And even with respect to ardent spirits, although they can only be regarded as diluted alcohol, still each species appears to possess a peculiarity of operation; owing, no doubt, to the modifying influence of the other elements of the liquid; thus *brandy** is said to be cordial and stomachic; *rum* more heating and sudorific; *gin* and *whiskey*, diuretic; and *arrack*, styptic, heating, and narcotic. It seems also that a modified effect is produced

---

* "I apprehend that the peculiar flavour of *cogniac* depends upon the presence of an æthereal spirit, formed by the action of tartaric, or perhaps acetic acid upon alcohol. It is on this account that nitric æther, when added to malt spirits gives them the flavour of brandy." *Pharmacologia, vol.* 2, p. 396.

by the addition of various other substances, such as sugar and acids; which latter bodies, besides their anti-narcotic powers, appear to act by favouring a more perfect combination and mutual penetration of the particles of spirit and water. The effects also which are produced by the habitual use of fermented liquors differ essentially according to the kind that is drunk; thus, ale and porter, in consequence of the nutritive matter, and perhaps the invigorating bitter with which they are charged, and the comparatively small proportion of alcohol which they contain, dispose to plethora, which is sometimes terminated by apoplexy." 436.

Alcohol in large and poisonous doses appears to destroy the functions of the brain, without occasioning that previous stage of excitement induced by smaller doses—whence coma and insensibility are the immediate consequences, and the patient dies apoplectic for want of the respiratory functions. Mr. Brodie was the first to propose artificial breathing in such cases; and in an experiment carefully performed on an animal under such circumstances he preserved its life. In most cases, however, the life of man (the only animal addicted to dram-drinking) is destroyed by accidents or contingencies, as exposure to cold, or suffocation from an imperfect act of vomiting, during which a portion of the contents of the stomach are forced into the trachea.

The action of alcohol on the brain is supposed by Mr. Brodie and our author to be sympathetic through the medium of the nerves.

The treatment is by emetics, bleeding, cold lotions to the head—easy position of the body, and a mild atmosphere.

The two remaining short sections on animal and aerial poisons contain nothing that we deem it necessary to lay before our readers. We have now finished the second volume of the work before us. About a fourth of the third or last volume, (not filled with acts of parliament, charters, trials, &c.) is on sudden and mysterious deaths, and infanticide. This portion will furnish us with matter for a fourth and last analytical paper.

